# The Elderly's Demand for Community-Based Care Services and Its Determinants: A Comparison of the Elderly in the Affordable Housing Community and Commercial Housing Community of China

**DOI:** 10.1155/2020/1840543

**Published:** 2020-10-21

**Authors:** Tiantian Gu, Dezhi Li, Lingzhi Li

**Affiliations:** ^1^School of Mechanics and Civil Engineering, China University of Mining and Technology, Xuzhou, Jiangsu 221116, China; ^2^School of Civil Engineering, Southeast University, Nanjing, Jiangsu 211189, China; ^3^School of Civil Engineering, Nanjing University of Technology, Nanjing, Jiangsu 211800, China

## Abstract

With the rapid aging of the world population, great pressure has been placed on the provision of community-based care in China. This paper aimed to compare the demand and its determinants for various community-based care services among the elderly in the affordable housing community (AHC) and commercial housing community (CHC) of China. Two community-based surveys were conducted separately in the AHC and CHC of Nanjing City, China. In total, 408 valid questionnaires were returned from the Daishan AHC while 8422 valid questionnaires were received from the CHCs. The chi square test indicated that the respondents in the AHC had significantly higher demands for five types of services (the meal-aid service, the cleaning-aid service, the bath-aid service, the rehabilitation therapy service, and the first-aid service) than those in the CHCs of Nanjing. Further, the Cochran–Mantel–Haenszel test showed that factors influencing the elderly's demands for these services varied across communities. Several policy implications could be obtained to improve the efficiency of community-based care provision.

## 1. Introduction

As the proportion of the world's population over 60 years old is increasing, the number of the elderly in need of health and social care is growing. Considering the imbalance between care demand and supply, community-based care has been advocated and became an increasingly important mode of care provision in many societies. Due to different welfare regimes, economies, and cultures, the concept of community-based care is likely to differ in various ways. For instance, in western countries, the elderly desire to get a variety of elderly care services in their own homes, also known as “aging in place” [1]. Correspondingly, “community-based care” is used in policy terms of China, and it is defined as professional care provided by government and social forces to the elderly within their locality [2– 4]. Although the rise of community-based care provides a feasible solution to narrow the imbalance [5, 6], challenges of the elderly care service supply for both policy and practice are burned, such as how to know the actual demand of the elderly for different types of community-based care and how to provide services for the elderly with different demands efficiently [4, 7].

In China, the speed of population aging exceeds the speed of economic and social development, which leads to the slow progress of community-based care [8, 9]. By the end of 2019, the proportion of people aged 60 years or over in China had reached 18.10% [10]. Considering resource constraints, the provision of community-based care is seriously insufficient in the commercial housing community (CHC), which is a common type of urban settlements developed by the real estate development companies with the approval of the relevant government departments for sale or rent in the market [11, 12]. In contrast with the CHC, the shortages of elderly care service supply are more acute in the affordable housing community (AHC) of China. Generally, the AHC was vigorously advocated from 2011 to 2015 to resolve the growing housing problems for low-income people in China [13, 14]. Due to the innate characteristics of the remote location, high aging rate, and low-income population aggregation, the demand for community-based care services in the AHC is increasing rapidly while the supply of these services is seriously inadequate [4, 15]. Consequently, elderly care-related issues have been raised, such as single service supply content, inefficiency of allocation of elderly care resources, unmatched service requirements, and low satisfaction with the elderly care services [16].

Much of the literature on aging issues in urban communities is extensive and focuses particularly on indoor facilities management [17], assessing elderly adaptability [18], use of formal and informal sources of mental health care [19], the allocation of elderly healthcare facilities [11, 20], demand for public space in elderly housings [21], the elderly's demand for community-based care services in the AHC [22], etc. In particular, the demands for the elderly care hotline, building health archives, on-call nursing and doctor visits, regular medical examinations, and sporting fitness among the elderly in the AHC have been adequately explored.

However, previous studies considered urban communities as a whole without considering the characteristics of the elderly in different communities, which led to some challenges in this research field. First, as the main type of urban communities, limited attention has been paid to the aging problem in the CHC [23, 24]. In more detail, there has been little systematic analysis available about how to analyze the demands of the elderly in the CHC for various types of community-based care services and the influencing factors related to the demand side. Second, little research has been conducted to quantitatively compare the difference of the elderly's demands for community-based care services between the AHC and the CHC. Third, up to this point, few studies have systematically compared the influencing factors of their demands for these services in both communities or explored whether there are specific issues that should be considered in each environment, respectively.

In light of the severe aging population in the whole world, especially in China, understanding the demand of the elderly for community-based care services in different types of communities and its determining factors is vital for offering new insights into the development of community-based care in order to clarify different elderly's service demands and enhance the efficiency of community-based care provision [25]. Moreover, this study is of great significance in compensating for the deficiency of the previous qualitative and descriptive analysis. Since the elderly themselves are most qualified to give a comprehensive view of their perceived care demands, it is vital to listen to the demand of the elderly in the AHC and the CHC separately and take their characteristics into account in the care provision [26, 27]. Hence, this study focuses on the elderly in these two communities, and it has three aims:

1. To quantify and compare the elderly's demands for different types of community-based care services in the AHC and the CHC of China.
2. To examine and compare determinants of the elderly's demand for these services in both communities.
3. To provide suggestions about improving the efficiency of community-based care provision.

## 2. Literature Review

The context for our analysis should be provided by introducing community-based care services. It is generally recognized that community-based care services cover a variety of social and health services and it is ambiguous in that the development of such services is adapted to the particular national and cultural conditions [28, 29]. Thus, guided by existing literature, community-based care services for the elderly can be divided into four categories (i.e., assistance with activities of daily life service, medical care service, cultural and entertainment service, and psychological and legal service), and each category of these services includes different types of services [22]. Previous literature also suggests that six types of services, namely, meal-aid service (MAS), cleaning-aid service (CAS), bath-aid service (BAS), rehabilitation therapy service (RTS), first-aid service (FAS), and daily mental care service (DMCS), were predominantly far from meeting the elderly's demand [2, 3,22, 30–34]. The specific contents of these services are presented in Table 1.

Regarding the relevant research studies on the influencing factors of elderly's demands for community-based care services, many scholars have explored this field to a large extent. Examples of individual-level factors that affect these demands include demographic structure (e.g., age and gender), family structure (living status), income, and elderly care intention [2, 30, 35–38]. Specifically, as for the demographic structure, several lines of evidence suggest that more than 50% of the elderly were likely to have demand for medical services, and men have higher demand for such services [39, 40]. In terms of the family structure, old people who do not live alone were more willing to obtain various care services from their community [41]. As for income, it has been demonstrated that the elderly with higher monthly income were more likely to have a demand for on-call nursing and doctor visits [35]. In addition, previous studies have found that older people who prefer to get the elderly service in long-term care institutions had a higher demand for the RTS [35, 42]. It is also notable that the influence of age, education level, and family structure on the demands of the elderly in the AHC was not obvious, while it had a significant influence on the demands of the elderly in urban communities [35, 42]. Overall, these studies illustrate that different factors could influence the elderly's demands for community-based care services, and the determinant varies across different types of service and environmental settings.

The aforementioned findings have made substantial progress in community-based care research and served as feedback to policy makers. However, such studies remain narrow in dealing only with the classification of elderly care service demands and analysis on the elderly's service demand in specific areas. Hitherto, differences in the elderly's demands for community-based care services in different communities and determinants of these demands are barely compared in the literature.

## 3. Methods

**Figure 1:**
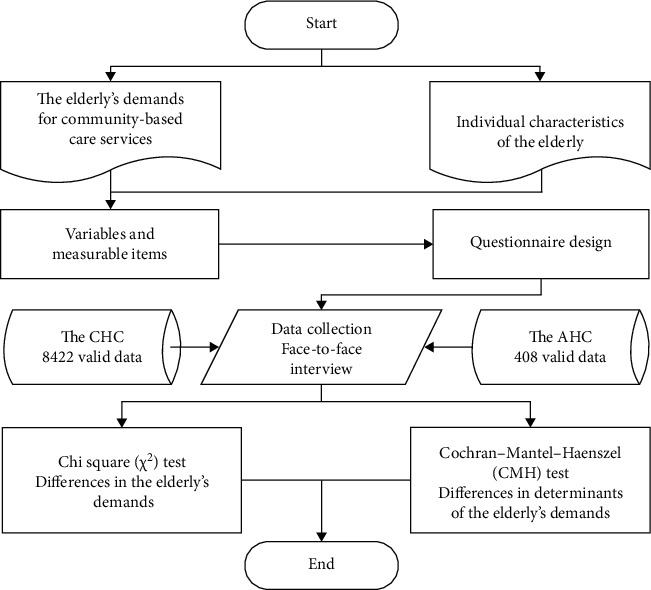
The whole flowchart of our study protocol.

In order to quantitatively analyze the differences between the demands of the elderly for community-based care services and determinants of these demands in the AHC and the CHC of China, variables and measures were determined, and the questionnaire was designed. Then, two quantitative surveys were developed in the CHC and the AHC separately to collect related data. Finally, the chi square (*χ*^2^) test and Cochran–Mantel–Haenszel (CMH) test were applied for data analysis. The whole flowchart of the study protocol is shown in Figure 1.

### 3.1. Variables and Measures

Considering there are currently no standard validated surveys analyzing the elderly's demand for community-based care services and its determinants, a structured questionnaire (encompassed six close-ended questions) was developed to obtain the relevant information.

As for the measurement of the elderly's demand for community-based care services, the question “Do you need this type of community-based care services ?” was used. The response variable was measured as a dichotomous variable with possible values 0, if the respondent needed this type of community-based care services and 1 otherwise. It is known from the findings of our literature review that the elderly generally have higher demand for the MAS, the CAS, the BAS, the RTS, the FAS, and the DMCS [2, 3, 22, 30]. Thus, these six types of community-based care services were selected as outcome variables and they were measured, respectively, by responses to the question above.

According to the prior work, individual characteristics have been reported to be correlated with determining the elderly's demand for different types of community-based care service, such as sociodemographic characteristics, family structure, economic characteristics, and elderly care intention. Considering the second aim of this study and the feasibility of large sample data collection, the potential influencing factors include gender (1 = “male”; 2 = “female”), age (classified into 5 groups: 1 = “60–64”; 2 = “65–69”; 3 = “70–74”; 4 = “75–79”; 5 = “ ≥80”), living alone or not (0 = “no”; 1 = “yes”), monthly income(classified into 3 groups: 1 = “<1000”; 2 = “1000–3000”; 3 = “ >3000”), and elderly care intention (1 = “private home”; 2 = “institution”; 3 = “community facilities”) [22].

### 3.2. Sampling and Data Collection

This research was based on data obtained from a large-scale investigation conducted in the CHCs of Nanjing from August 2017 to April 2018 and a cross-sectional field survey conducted in the Daishan AHC of Nanjing in May, 2018. The study areas were selected for two reasons. On the one hand, Nanjing is one of the ten most suitable old-age cities in China and it has a higher development level of elderly care services [43]. On the other hand, Daishan AHC in Nanjing is typical in in terms of the aggregation of the low-income elderly population and urgent demand for community-based care service [11]. In the process of conducting both investigations, random sampling and the face-to-face interview method were used to ensure high response rates and reliable results.

With the help of the Nanjing Civil Affairs Bureau, more than 11,724 questionnaires were distributed to the elderly people over 60 years old by many volunteers in the vast majority of the CHCs of Nanjing and 8422 valid data were received (the rate of effective recovery was 71.84%). Meanwhile, to achieve the statistical theoretical sample size and decrease multiple deviations in the survey of the Daishan AHC, fourteen trained postgraduates from our research group of Southeast University participated in the interview with 420 elderly people living in the Daishan AHC [22]. 408 valid data were collected after the face-to-face interview (the rate of effective recovery was 97.14%). Finally, data on 8422 elderly people from the CHCs and 408 elderly people from the Daishan AHC in Nanjing were collected (8830 elderly people in total).

### 3.3. Statistical Analysis

Most of the previous research studies applied the chi square (*χ*^2^) test for all bivariate analyses to compute the comparison of proportions across outcome variables between two groups [44, 45]. Due to the characteristic of the outcome variables (categorical variable): coded 0 (need) or 1 (no need), the *χ*^2^ test was used to test the differences between the elderly from the AHC and the CHC in their demand for six types of community-based care services, respectively. Additionally, the Cochran–Mantel–Haenszel (CMH) test is often used when controlled covariates affect the effect of explanatory variables on outcome variables [46]. In this study, the impact of individual characteristics on the elderly's demand may vary with the type of community. In order to compare the differences in those impacts across the two types of communities, the CMH test was applied to summary data obtained from the respondents and the elderly in the CHC was used as the reference group in the analyses. Data processing was performed with SPSS 22.0 statistical software. Then, 95% confidence intervals (95% CI) and odds ratios (OR) were obtained by computing the odds.

## 4. Results

The surveys collected information about respondents' sociodemographic characteristics, family structure, economic characteristics, elderly care intention, and their demands for six types of community-based care services in the Daishan AHC and the CHCs of Nanjing. Analysis of the information was performed below.

### 4.1. Descriptive Statistics of the Respondents

**Figure 2:**
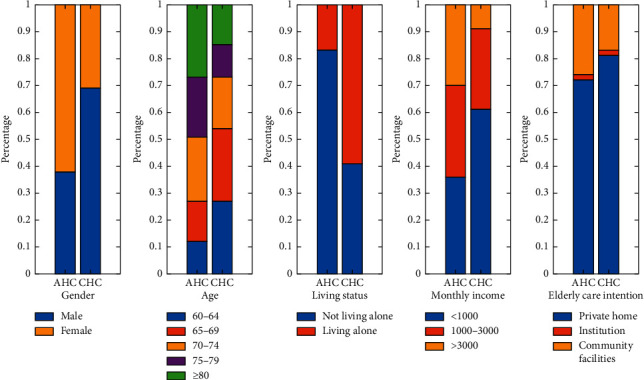
Simple descriptive statistics of the respondents in the AHCs and CHCs.

Figure 2 presents individual characteristics of the respondents in both Daishan AHC and the majority of the CHCs in Nanjing. In terms of sociodemographic characteristics, the respondents in the Daishan AHC were predominately female and belonged to higher age group (older than 70 years old) while the respondents in the CHCs were predominately male and young elderly people (60–74 years old). As for living status, 83.33% of the respondents in the Daishan AHC lived with others while only 41.17% of the respondents in the CHCs lived with others. In terms of economic characteristics, the number of people in the Daishan AHC whose monthly income was less than 1000 yuan accounts for 35.80%, followed by the number of people whose income was 1000–3000 yuan (34.31%). Surprisingly, 61.54% of the respondents in the CHCs had lower monthly incomes (less than 1000 yuan) and only 8.82% of the respondents had a higher monthly income (more than 3000 yuan), which means that the income polarization of the respondents in the CHCs is more severe than that in the Daishan AHC. In terms of elderly care intention, private home was the choice for 72.1% of the respondents in the Daishan AHC, community-based care facilities were the choice for 26.5% of the respondents, and only 1.5% of the respondents wanted to get elderly services in long-term care institutions. Similarly, respondents in the CHCs were more willing to get elderly services in private home. Care at home and care in the community are still the most ideal choice for the elderly in AHCs and CHCs, with the institutional care being the least favorable. The survey outcome is consistent with actual situation.

### 4.2. Differences in the Elderly's Demand for Community-Based Care Services

The comparison between the AHC and the CHC in terms of the elderly's demand for community-based care services conducted using *χ*^2^ test is shown in Table 2. Except for the elderly's demand for the DMCS (*χ*^2^=0.067, *p* > 0.05), there were statistically significant differences in the elderly's demand for various services. So, the DMCS is not considered in this analysis.

It was indicated that the respondents in the Daishan AHC had higher demands for five types of services with significant differences than those in the CHCs of Nanjing, and demands for the MAS and FAS varied the most (reaching more than 20%). It is noteworthy that although the proportion of the BAS demand was the smallest in the Daishan AHC (21.74%), it was higher than that of the demand for the MAS (21.38%), the FAS (21.07%), the CAS (19.29%), and the BAS (9.12%) in the CHCs. Specifically, the respondents of these two types of communities needed the RTS most (50.00% for the AHC and 43.61% for the CHC), while their demand for the BAS was the least (21.74% for the AHC and 9.12% for the CHC). Further, the RTS, the FAS, and the MAS are three most demanded services for the elderly in both communities, which reflects their strong willingness to access these services. In other words, physiological needs and safety needs of the elderly have not been fully met.

### 4.3. Differences in Factors Associated with the Elderly's Demand for the MAS

In the CMH test of factors related to the elderly's demand for the MAS, their demands are classified by community types. Test results are shown in Table 3. According to this table, gender, age, living status, monthly income, and elderly care intention were significantly associated with the elderly's demand for the MAS. Except for monthly income (*p* value of homogeneity test > 0.05), substantial heterogeneity of odds ratio existed after stratification for these factors.

Specifically, the community type was a significant correlate of the elderly's demand for all men (CMH *χ*^2^=62.467, *p* < 0.001) and women (CMH *χ*^2^=34.676, *p* < 0.001). Older adults (both men and women) from the AHC had higher demands for the MAS than the elderly from the CHC (OR = 3.412, 95% CI 2.473 to 4.709 and OR = 2.177, 95% CI 1.672 to 2.834, respectively). Of the respondents, those aged 70–74 years, 75–79 years, and  ≥ 80 years in the AHC were 4.909, 2.682, and 3.236 times more likely to have such demand than those in the CHC. However, community type was not significantly associated with the elderly's demand for other age groups. Once again, compared with the elderly in the CHC, respondents who lived with others in the AHC and those lived alone in the AHC were found to be 2.503 times and 8.012 times more likely to have this demand. Although no significant difference exists between all income groups, significantly higher percentage of the respondents in the AHC reported the demand for the MAS than those in the CHC (MH OR = 2.383, *p* < 0.001). Conversely, while respondents in the AHC who preferred to obtain the MAS in the private home and community-based care facility were 2.138 times and 6.720 times as likely as those in the CHC, the community type did not significantly predict the elderly's demand for the elderly who preferred to get elderly care in long-term care institution.

### 4.4. Differences in Factors Associated with the Elderly's Demand for the CAS

Similar to the findings for respondent's demand for the MAS, the elderly's demand for the CAS was significantly associated with gender, age, living status, monthly income, and elderly care intention (Table 4). There was significant statistical heterogeneity among age groups and monthly income groups (both *p* values of homogeneity test > 0.05).

According to Table 4, no significant heterogeneity between gender groups was present. For all the elderly (both men and women), those from the AHC were 1.762 times (MH OR = 1.762, *p* < 0.001) more likely to report the CAS demand than those from the CHC. If gender is not taken into account, the impact of the community type on the CAS demand is likely to be overestimated (OR = 1.915 > MH OR = 1.762). Compared with the respondents in the CHC, those aged 70–74 years and 75–79 years in the AHC had higher demands for the CAS (OR = 3.502, 95% CI 2.293 to 5.349 and OR = 2.123, 95% CI 1.341 to 3.359, respectively). Although the community type was significantly associated with the elderly's demand for the CAS when stratified by living status (CMH *χ*^2^=35.848, *p* < 0.001), there were no significant differences between both groups (*p* value of homogeneity test > 0.05). Further, respondents in the AHC were 1.998 times (MH OR = 1.998, *p* < 0.001) more likely to have demands for the CAS than those in the CHC after accounting for the effects of living status. Conversely, while those with monthly income of 1000–3000 yuan and more than 3000 yuan in the AHC were 2.569 and 5.852 times as likely as those in the CHC to report the CAS demand, the community type did not significantly predict such demand for the respondents with the monthly income of less than 1000 yuan. Considering the stratification factor of the elderly care intention, significantly higher percentages of the respondents in the AHC reported the CAS demand (MH OR = 1.794, *p* < 0.001).

### 4.5. Differences in Factors Associated with the Elderly's Demand for the BAS

According to Table 5, the CMH test result of the BAS demand was similar to the CAS demand. All stratification factors influence the effects of community types on the BAS demand, and significant statistical heterogeneity among age groups and monthly income groups existed.

Compared with the respondents in the CHC, all the elderly (both men and women) in the AHC were 2.582 times more likely to have the BAS demand (MH OR = 2.582, *p* < 0.001). Differently, there was significant heterogeneity among age groups. Except for those aged 65–69 years, the respondents in the AHC had a higher demand for the BAS than those in the CHC, and the greatest difference was found for those aged 70–74 years. Besides, tests of homogeneity of the odds ratio for living status showed a *χ*^2^ value of 0.779 (*p* < 0.05) and tests of conditional independence for living status showed a *χ*^2^ value of 76.720 (*p* < 0.001), which means that respondents in the AHC were 2.908 times (MH OR = 2.908, *p* < 0.001) more likely to have demand for the BAS than those in the CHC when stratified by living status. As for the effect of monthly income, those with monthly income of less than 1000 yuan and more than 3000 yuan in the AHC were slightly more likely to have the BAS demand than those in the CHC (OR = 3.079, *p* < 0.05 and OR = 6.129, *p* < 0.05, separately). Considering the impact of the elderly care intention, there were no significant differences between the three groups and respondents in the AHC were 2.697 times (MH OR = 2.697, *p* < 0.001) more likely to have the BAS demand than those in the CHC.

### 4.6. Differences in Factors Associated with the Elderly's Demand for the RTS

Table 6 shows that the association between community types and the elderly's demand for the RTS was significantly influenced by all stratification factors. There was significant statistical heterogeneity among living status groups and monthly income groups.

In detail, respondents from the AHC were 1.312 times (MH OR = 1.312, *p* < 0.01) more likely to report the RTS demand than those from the CHC when gender was included as a stratification variable. This finding held when the analyses were conducted by age. Slightly higher percentage of respondents in the AHC reported the demand for the RTS than those in the CHC (MH OR = 1.236, *p* < 0.05). Conversely, those lived alone in the AHC were 2.626 times (OR = 2.626, *p* < 0.01) more likely to have the RTS demand than those in the CHC while the effect of those living with others on relationship between community types and the RTS demand was not significant. Considering monthly income as a stratification factor, those with monthly income more than 1000 were significantly more likely to have the RTS demand than those in the CHC. Finally, when stratified by elderly care intention, respondents in the AHC were 1.235 times more likely to have higher demands for the RTS than those in the CHC (MH OR = 1.235, *p* < 0.05).

### 4.7. Differences in Factors Associated with the Elderly's Demand for the FAS

As shown in Table 7, the effects of community types on the FAS demand were influenced by all stratification factors and significant statistical heterogeneity existed among gender groups and age groups.

To be more specific, based on the CMH test result of gender, elderly males and elderly females in the AHC were 4.224 and 2.775 times more likely to report the FAS demand than those in the CHC. Similarly, compared with the reference group, the percentage of all age groups with the FAS demand in the AHC is slightly higher. In addition, when living status, monthly income, and elderly care intention were included as stratification factors, respondents in the AHC were 3.199, 2.887, and 3.085 times (MH OR = 3.199, *p* < 0.001, MH OR = 2.887, *p* < 0.001, and MH OR = 3.085, *p* < 0.001, respectively) more likely to have the FAS demand than those in the CHC.

### 4.8. Differences in Factors Associated with the Elderly's Demand for the DMCS

According to Table 8, the influence of community types on the respondents' demand for the DMCS was not significant when all stratification factors were considered, which is consistent with our finding of differences in the elderly's demand for community-based care services. Notably, although the partial results of *χ*^2^ test for age, living status, monthly income, and elderly care intention were significant, tests of homogeneity of the odds ratio and tests of conditional independence for those variables showed the *p* value > 0.05, which means that the association between community types and the DMCS demand was not likely to be affected by those variables.

## 5. Discussion

By using the *χ*^2^ test and CMH test to analyze the survey data, the elderly's demand and its determinants in the AHC and CHC were quantified and compared. We can be confident of some main findings.

First, it is shown that the elderly's service demands in the AHC are generally higher than those in the CHC (except the demand for DMCS). This finding is almost similar to the studies of Cai and Ao [35], Wu et al. [40], and Zhang and Li [47]. Generally, the CHC is better equipped with many public facilities (e.g., restaurants, hospitals, and public baths), where the elderly have more access to common services like most adults, while the AHC is far away from the urban business district and the public facilities are not perfect compared with the CHC [11]. This implies, still, that the elderly in the AHC experience more social isolation than other groups and getting access to community-based care is not easy for them [11, 48, 49]. Besides, the proportion of the elderly in the AHC is far more than that in the CHC, which leads to a relatively high demand for elderly care services in the AHC [22]. Thus, a priority for elderly care in the future should be the provision of community-based care for the elderly in the AHC. Moreover, as the “only child” generation age, the elderly's demand forcommunity-based care in the CHC will be increased gradually [4]. Notably, the elderly's demand for the DMCS was not statistically associated with community types. This result is supported by the studies of Zhu et al. and Chen et al. [39, 50].

Second, in terms of sociodemographic characteristics, there are gender and age differences in the elderly service demands-community types across cohorts (except the demand for the DMCS). Specifically, for the MAS and the FAS, the relationships between elderly's demands for these services and community types are stronger among elderly males than among elderly females. One possible explanation is that the elderly males are more likely to have poor living ability and they care more about diet and first aid [51]. Similarly, age differences between the elderly in both communities could also affect the analysis result for the MAS, the CAS, the BAS, and the FAS. Compared with the elderly in the CHC, older people aged 70–74 in the AHC had the highest reported need for the MAS, the CAS, and the BAS. This result is not surprising because the elderly in higher age groups (70 years and older) generally suffered from multiple diseases and are unable to engage in self-care [52]. Services related to diet and personal cleaning have naturally become more popular among this age group. In the future, the increase in the number of the elderly aged 70 and over will bring more service demand [4].

Third, in terms of family structure, living status was statistically associated with differences in the elderly's demand for the MAS and the RTS between two types of communities. Precisely, compared with the elderly living with others, the elderly who lived alone have a greater impact on differences in the MAS and the RTS demand between the AHC and the CHC. Moreover, the elderly who lived alone are more likely to have a demand for the RTS, while this effect did not work for the elderly who lived with others. With the development of social economy and the implementation of the family planning policy, weaker family supports and intergenerational relationships are gradually formed, especially in the AHC. Consequently, there are more and more elderly people living alone in communities, and they need external sources to obtain elderly care services, especially meal-aid service and rehabilitation therapy service [4, 29]. In addition, differences in the elderly's demand for the CAS, the BAS, and the FAS between the AHC and the CHC were not statistically related to living status. It may be because a large number of young people migrate to work under the influence of urbanization, modernization, and the weakening of filial piety [22]. As a result, many elderly people are less likely to receive care from their children. Whether the elderly live alone or not, only seeking personal cleaning service and emergency service could guarantee their quality of life.

Fourth, as an important economic characteristic, monthly income does have a significant moderating effect on the relationship between community types and the elderly's demand for the CAS, the BAS, and the RTS. In more concrete terms, better off older people (monthly income of more than 3000 yuan) have comparatively high demands for the CAS and the BAS, while middle-income older people (monthly income of 1000–3000 yuan) in the AHC had the highest reported need for the RTS compared with those in the CHC. One possible explanation for this phenomenon is that the more affluent tend to be more demanding than the less well-off [4]. However, there is no difference between various income groups in the elderly's demand for the MAS and the FAS between both communities. Considering that the physiological needs and safety needs are the fundamental need for the elderly according to Maslow's hierarchy of needs, their demands for these services are increasing, whether they are poor or rich [53].

Fifth, elderly care intention shows mediator roles in the relationship between community types and elderly's demand for the MAS. Specifically, among the subgroups with the demand for the MAS, the elderly in the AHC who preferred to receive care from the private homes or community-based care facilities are more likely to have a higher service demand than those in the CHC. Despite being a family-oriented country, care from family members did not reduce the demand for community-based care and even seems to increase it. This effect is more obvious in the AHC [4]. As a result, older people in the AHC who are willing to get services in private homes or community-based care facilities hold more positive attitudes towards the MAS than those in the CHC [54]. Additionally, the elderly care intention may not play a moderating role in the association of community types and elderly's demand for the CAS, the BAS, the RTS, and the FAS. Presumably, this is because the differences of elderly's demands for these services between the AHC and the CHC are so pronounced that any possible moderating effect is cancelled.

## 6. Conclusion

As the number of older people increases, many of them are characterized by unhealthy longevity. China's nascent community-based care system is facing severe challenges. Substantial demands for social and health services among the elderly in both the AHC and the CHC have been formed. This paper aims to generate fresh information about the subjective demands for six types of community-based care services among the elderly in both communities. The *χ*^2^ test and CMH test were applied to 8830 data from two types of communities of Nanjing to explore and compare the elderly's demand for community-based care services and its determinants. The in-depth analysis results reveal that the respondents' demands for the MAS, the CAS, the BAS, the RTS, and the FAS in the AHC were significantly higher than those in the CHC. Besides, the differences in the elderly's demands for these services between two types of communities were influenced by distinct factors. The result helps to clarify the difference in the demand for the community-based care among the elderly in both communities and compensate for the deficiency of the previous qualitative and descriptive analysis. More importantly, it might promote the formation of effective integrated social systems of community-based care for the elderly in different communities. We believe that this type of analysis has been done in the context of the community-based care for the first time.

To promote better coordination of services, several policy implications can be obtained according to these empirical results. First, more attention and resources should be paid to the elderly in the AHC. It is essential to increase subsidies to social organizations for the aged in the AHC to ensure that as many community-based care services as possible are offered in such community. Second, it is a practical way to introduce the market mechanism in the CHC to improve the community-based care system and make up for the lack of elderly services caused by government intervention. Third, it is suggested that an integrated information platform could be established to collect the data related to the needs of certain elderly subgroups. By considering individual characteristics of the elderly, it is possible for the elderly and their relatives to choose customized services. Additionally, precise financial supports for low-income elderly could be provided to enhance their purchasing power for elderly care services. Finally, publicity for community-based care system and the notion of “respect and care for the elderly” should be given to encourage various social forces (e.g., friends, volunteers, neighbors, and social institutions) to participate in the provision of community-based care services. However, due to the relatively small sample size of the AHC in this study, insignificant results need to be explained with care. Future research will consider a similar examination of the elderly' demand with different types of community-based care services in other AHCs of China. Larger-scale sets of data will be used when data become available.

## Figures and Tables

**Table 1:** A summary of six types of community-based care services with high demand.

Category	Type	Brief description	Researchers
Assistance with activities of daily living service	1. Meal-aid service (MAS)	Providing canteens or centralized meal delivery service.	Zhu, 2017; Wang, 2013; Gu et al., 2020; Shang, 2014.
	2. Cleaning-aid service (CAS)	Providing indoor cleaning services and specialized cleaning services for the elderly.	
	3. Bath-aid service (BAS	Providing visiting bath service for the elderly to help them take a bath.	

Medical care service	4. Rehabilitation therapy service (RTS)	Providing care that can help the elderly get back, keep, or improve abilities that they need for daily life. These abilities may be physical, mental, and/or cognitive.	Zhu, 2017; Hicks et al., 2015; Rekha, et al., 2017.
	5. First-aid service (FAS)	Providing emergency responses to unexpected serious health events and unexpected security incidents, such as offering assistance to the elderly suffering from sudden cardiovascular and cerebrovascular diseases.	

Psychological and legal service	6. Daily mental care service (DMCS)	Providing relief to mental disorders and alleviating mental stress for the elderly to meet their daily mental needs.	Zhu, 2017; Wang, 2013; Strasser et al., 2013.

**Table 2:** Comparison of the elderly's demand for services with two types of communities.

Type of services	*N*	The AHC (*N* = 408 (%)	The CHC (*N* = 8422 (%)	*χ* ^2^
*The MAS*				
Need	1983	2.00	20.40	115.246^*∗∗∗*^
No need	6847	2.60	75.00	
Need/total (%)	22.46%	43.48	21.38	

*The CAS*				
Need	1751	1.40	18.40	35.848^*∗∗∗*^
No need	7079	3.20	77.00	
Need/total (%)	19.83%	30.43	19.29	

*The BAS*				
Need	852	1.00	8.70	69.715^*∗∗∗*^
No need	7978	3.60	86.70	
Need/total (%)	9.65%	21.74	9.12	

*The RTS*				
Need	3879	2.30	41.60	6.299^*∗∗*^
No need	4951	2.30	53.80	
Need/total (%)	43.93%	50.00	43.61	

*The FAS*				
Need	1958	2.10	20.10	135.886^*∗∗∗*^
No need	6872	2.50	75.30	
Need/total (%)	22.17%	45.65	21.07	

*The DMCS*				
Need	3430	1.80	37.10	0.067
No need	5400	2.90	58.30	
Need/total (%)	38.84%	38.30	38.89	

*Note*. Significance level: ^*∗*^*p* < 0.05, ^*∗∗*^*p* < 0.01, and ^*∗∗∗*^*p* < 0.001.

**Table 3:** CMH test of factors associated with the elderly's demand for the MAS.

Factors		*χ* ^2^	OR (AHC/CHC)	95% CI	MH OR
Gender	Male	62.467^*∗∗∗*^	3.412^*∗*^	2.473–4.709	—
	Female	34.676^*∗∗∗*^	2.177^*∗*^	1.672–2.834	—
	Total	115.246^*∗∗∗*^	2.898	2.368–3.548	2.575

Age	60–64	1.55	1.515	0.784–2.925	—
	65–69	1.904	0.593	0.280–1.256	—
	70–74	66.421^*∗∗∗*^	4.909^*∗∗∗*^	3.238–7.442	—
	75–79	20.26^*∗∗∗*^	2.682^*∗∗∗*^	1.722–4.178	—
	≥80	36.088^*∗∗∗*^	3.236^*∗∗∗*^	2.169–4.827	—
	Total	115.246^*∗∗∗*^	2.898	2.368–3.548	2.603

Living status	No	66.304^*∗∗∗*^	2.503^*∗∗∗*^	1.994–3.142	—
	Yes	76.204^*∗∗∗*^	8.012^*∗∗∗*^	4.636–13.845	—
	Total	115.246^*∗∗∗*^	2.898	2.346–3.548	3.067

Monthly income	<1000	23.480^*∗∗∗*^	2.283	1.621–3.216	—
	1000–3000	15.609^*∗∗∗*^	2.738	1.629–4.602	—
	>3000	6.399^*∗∗∗*^	2.252	1.183–4.287	—
	Total	115.246^*∗∗∗*^	2.898	2.368–3.548	2.383^*∗∗∗*^

Elderly care intention	Private home	37.814^*∗∗∗*^	2.138^*∗∗∗*^	1.669–2.738	—
	Institution	0.219	0.664	0.118–3.730	—
	Community facility	95.143^*∗∗∗*^	6.720^*∗∗∗*^	4.373–10.325	—
	Total	115.246^*∗∗∗*^	2.898	2.368–3.548	2.858

*Note.* The elderly in the CHC served as the reference group. *p* values were calculated using stratified Cochran–Mantel–Haenszel tests, with sex, age, living status, monthly income, and elderly care intention group as stratification. Significance level: ^*∗*^*p* < 0.05, ^*∗∗*^*p* < 0.01, and ^*∗∗∗*^*p* < 0.001. OR, odds ratio; 95% CI, 95% confidence interval; MH OR, Mantel–Haenszel common odds ratio.

**Table 4:** CMH test of factors associated with the elderly's demand for the CAS.

Factors		*χ* ^2^	OR (AHC/CHC)	95% CI	MH OR
Gender	Male	10.798^*∗∗*^	1.799	1.261–2.566	—
	Female	15.628^*∗∗∗*^	1.741	1.319–2.299	—
	Total	35.848^*∗∗∗*^	1.915	1.543–2.377	1.762^*∗∗∗*^

Age	60–64	0.172	1.16	0.575–2.338	—
	65–69	0.029	0.942	0.475–1.871	—
	70–74	37.459^*∗∗∗*^	3.502^*∗∗*^	2.293–5.349	—
	75–79	10.725^*∗∗*^	2.123^*∗∗*^	1.341–3.359	—
	≥80	0.883	1.221	0.805–1.853	—
	Total	35.848^*∗∗∗*^	1.915	1.543–2.377	1.724

Living status	No	29.512^*∗∗∗*^	1.937	1.520–2.467	—
	Yes	11.196^*∗∗*^	2.27	1.387–3.716	—
	Total	35.848^*∗∗∗*^	1.915	1.543–2.377	1.998^*∗∗∗*^

Monthly income	<1000	0.132	0.923	0.601–1.419	—
	1000–3000	12.812^*∗∗∗*^	2.569^*∗∗∗*^	1.505–4.386	—
	>3000	32.867^*∗∗∗*^	5.852^*∗∗∗*^	2.991–11.448	—
	Total	35.848^*∗∗∗*^	1.915	1.543–2.377	1.762

Elderly care intention	Private home	16.621^*∗∗∗*^	1.746	1.331–2.290	—
	Institution	0.069	0.794	0.141–4.461	—
	Community facility	11.997^*∗∗∗*^	1.983	1.338–2.939	—
	Total	35.848^*∗∗∗*^	1.915	1.543–2.377	1.794^*∗∗∗*^

*Note.* The elderly in the CHC served as the reference group. *p* values were calculated using stratified Cochran–Mantel–Haenszel tests, with sex, age, living status, monthly income, and elderly care intention group as stratification. Significance level: ^*∗*^*p* < 0.05, ^*∗∗*^*p* < 0.01, and ^*∗∗∗*^*p* < 0.001. OR, odds ratio; 95% CI, 95% confidence interval; MH OR, Mantel–Haenszel common odds ratio.

**Table 5:** CMH test of factors associated with the elderly's demand for the BAS.

Factors		*χ* ^2^	OR (AHC/CHC)	95% CI	MH OR
Gender	Male	33.394^*∗∗∗*^	3.004	2.032–4.442	—
	Female	27.828^*∗∗∗*^	2.354	1.699–3.263	—
	Total	69.715^*∗∗∗*^	2.756	2.152–3.530	2.582^*∗∗∗*^

Age	60–64	10.148^*∗∗*^	3.004^*∗*^	1.477–6.110	—
	65–69	0.136	0.825	0.296–2.299	—
	70–74	45.824^*∗∗∗*^	4.517^*∗*^	2.816–7.248	—
	75–79	7.750^*∗∗*^	2.241^*∗*^	1.253–4.010	—
	≥80	10.325^*∗∗*^	2.056^*∗*^	1.314–3.215	—
	Total	69.715^*∗∗∗*^	2.756	2.152–3.530	2.449

Living status	No	52.198^*∗∗∗*^	2.739	2.062–3.638	—
	Yes	25.734^*∗∗∗*^	3.581	2.120–6.047	—
	Total	69.715^*∗∗∗*^	2.756	2.152–3.530	2.908^*∗∗∗*^

Monthly income	<1000	35.668^*∗∗∗*^	3.079^*∗*^	2.090–4.536	—
	1000–3000	0.235	1.235	0.525–2.905	—
	>3000	32.154^*∗∗∗*^	6.129*∗*	3.040–12.359	—
	Total	69.715^*∗∗∗*^	2.756	2.152–3.530	2.962

Elderly care intention	Private home	44.040^*∗∗∗*^	2.694	1.988–3.651	—
	Institution	0.042	1.198	0.212–6.760	—
	Community facility	23.228^*∗∗∗*^	2.903	1.850–4.556	—
	Total	69.715^*∗∗∗*^	2.756	2.152–3.530	2.697^*∗∗∗*^

*Note.* The elderly in the CHC served as the reference group. *p* values were calculated using stratified Cochran–Mantel–Haenszel tests, with sex, age, living status, monthly income, and elderly care intention group as stratification. Significance level: ^*∗*^*p* < 0.05, ^*∗∗*^*p* < 0.01, and ^*∗∗∗*^*p* < 0.001. OR, odds ratio; 95% CI, 95% confidence interval; MH OR, Mantel–Haenszel common odds ratio.

**Table 6:** CMH test of factors associated with the elderly's demand for the RTS.

Factors		*χ* ^2^	OR (AHC/CHC)	95% CI	MH OR
Gender	Male	9.689^*∗∗*^	1.656	1.202–2.282	—
	Female	0.798	1.126	0.868–1.459	—
	Total	6.399^*∗*^	1.292	1.059–1.576	1.312^*∗∗*^

Age	60–64	0.161	1.122	0.638–1.974	—
	65–69	0.893	1.275	0.769–2.113	—
	70–74	9.407^*∗∗*^	1.905	1.254–2.893	—
	75–79	0.053	0.95	0.613–1.474	—
	≥80	0.091	1.062	0.719–1.569	—
	Total	6.399^*∗*^	1.292	1.059–1.576	1.236^*∗*^

Living status	No	1.277	1.134	0.911–1.412	—
	Yes	14.382^*∗∗∗*^	2.626^*∗∗*^	1.563–4.378	—
	Total	6.399^*∗*^	1.292	1.059–1.576	1.301

Monthly income	<1000	0.005	0.988	0.710–1.374	—
	1000–3000	15.853^*∗∗∗*^	2.875^*∗∗*^	1.671–4.947	—
	>3000	3.895^*∗*^	1.888^*∗∗*^	0.995–3.580	—
	Total	6.399^*∗*^	1.292	1.059–1.576	1.41

Elderly care intention	Private home	2.801	1.221	0.966–1.543	—
	Institution	0.489	0.545	0.097–3.058	—
	Community facility	2.026	1.337	0.895–1.995	—
	Total	6.399^*∗*^	1.292	1.059–1.576	1.235^*∗*^

*Note.* The elderly in the CHC served as the reference group. *p* values were calculated using stratified Cochran–Mantel–Haenszel tests, with sex, age, living status, monthly income, and elderly care intention group as stratification. Significance level: ^*∗*^*p* < 0.05, ^*∗∗*^*p* < 0.01, and ^*∗∗∗*^*p* < 0.001. OR, odds ratio; 95% CI, 95% confidence interval; MH OR, Mantel–Haenszel common odds ratio.

**Table 7:** CMH test of factors associated with the elderly's demand for the FAS.

Factors		*χ* ^2^	OR (AHC/CHC)	95% CI	MH OR
Gender	Male	90.410^*∗∗∗*^	4.224^*∗*^	3.064–5.833	—
	Female	58.778^*∗∗∗*^	2.775^*∗*^	2.119–3.633	—
	Total	135.886^*∗∗∗*^	3.144	2.570–3.847	3.297

Age	60–64	42.327^*∗∗∗*^	5.439^*∗*^	3.081–9.599	—
	65–69	21.948^*∗∗∗*^	3.19^*∗*^	1.915–5.316	—
	70–74	44.086^*∗∗∗*^	3.742^*∗*^	2.476–5.657	—
	75–79	24.659^*∗∗∗*^	2.959^*∗*^	1.897–4.614	—
	≥80	8.1^*∗∗*^	1.801^*∗*^	1.196–2.714	—
	Total	135.886^*∗∗∗*^	3.144	2.570–3.847	2.992

Living status	No	119.1^*∗∗∗*^	3.254	2.605–4.065	—
	Yes	20.840^*∗∗∗*^	2.956	1.818–4.806	—
	Total	135.886^*∗∗∗*^	3.144	2.570–3.847	3.199^*∗∗∗*^

Monthly income	<1000	29.694^*∗∗∗*^	2.474	1.768–3.463	—
	1000–3000	39.126^*∗∗∗*^	4.54	2.708–7.609	—
	>3000	7.748^*∗∗*^	2.539	1.291–4.995	—
	Total	135.886^*∗∗∗*^	3.144	2.570–3.847	2.887^*∗∗∗*^

Elderly care intention	Private home	93.822^*∗∗∗*^	3.078	2.426–3.905	—
	Institution	0.001	1.009	0.179–5.684	—
	Community facility	38.874^*∗∗∗*^	3.329	2.240–4.947	—
	Total	135.886^*∗∗∗*^	3.144	2.570–3.847	3.085^*∗∗∗*^

*Note.* The elderly in the CHC served as the reference group. *p* values were calculated using stratified Cochran–Mantel–Haenszel tests, with sex, age, living status, monthly income, and elderly care intention group as stratification. Significance level: ^*∗*^*p* < 0.05, ^*∗∗*^*p* < 0.01, and ^*∗∗∗*^*p* < 0.001. OR, odds ratio; 95% CI, 95% confidence interval; MH OR, Mantel–Haenszel common odds ratio.

**Table 8:** CMH test of factors associated with the elderly's demand for the DMCS.

Factors		*χ* ^2^	OR (AHC/CHC)	95% CI	MH OR
Gender	Male	0.032	0.971	0.7–1.346	—
	Female	0.001	0.995	0.763–1.299	—
	Total	0.067	0.973	0.793–1.194	0.907

Age	60–64	0.064	0.927	0.517–1.663	—
	65–69	4.588^*∗*^	0.538	0.303–0.957	—
	70–74	5.501^*∗*^	1.624	1.079–2.442	—
	75–79	1.661	0.737	0.463–1.174	—
	≥80	0	0.997	0.669–1.484	—
	Total	0.067	0.973	0.793–1.194	0.961

Living status	No	4.037^*∗*^	0.789	0.626–0.995	—
	Yes	16.338^*∗∗∗*^	2.723	1.644–4.509	—
	Total	0.067	0.973	0.793–1.194	0.985

Monthly income	<1000	1.078	0.834	0.592–1.175	—
	1000–3000	18.137^*∗∗∗*^	3.01	1.770–5.121	—
	>3000	1.584	1.502	0.794–2.839	—
	Total	0.067	0.973	0.793–1.194	1.26

Elderly care intention	Private home	10.124^*∗∗*^	0.66	0.510–0.854	—
	Institution	3.514	1.598	1.419–1.799	—
	Community facility	15.648^*∗∗∗*^	2.208	1.479–3.297	—
	Total	0.067	0.973	0.793–1.194	0.956

*Note.* The elderly in the CHC served as the reference group. *p* values were calculated using stratified Cochran–Mantel–Haenszel tests, with sex, age, living status, monthly income, and elderly care intention group as stratification. Significance level: ^*∗*^*p* < 0.05, ^*∗∗*^*p* < 0.01, and ^*∗∗∗*^*p* < 0.001. OR, odds ratio; 95% CI, 95% confidence interval; MH OR, Mantel–Haenszel common odds ratio.

## Data Availability

All relevant data used to support the findings of this study have not been made available because these data were supplied by the Nanjing Civil Affairs Bureau of China under license.

## Abbreviations

AHC: Affordable housing community
CHC: Commercial housing community
MAS: Meal-aid service
CAS: Cleaning-aid service
BAS: Bath-aid service
RTS: Rehabilitation therapy service
FAS: First-aid service
DMCS: Daily mental care service.
